# Cellular Mechanisms of High Mobility Group 1 (HMGB-1) Protein Action in the Diabetic Retinopathy

**DOI:** 10.1371/journal.pone.0087574

**Published:** 2014-01-31

**Authors:** Andrea Rachelle C. Santos, Galina Dvoriantchikova, Yiwen Li, Ghulam Mohammad, Ahmed M. Abu El-Asrar, Rong Wen, Dmitry Ivanov

**Affiliations:** 1 Bascom Palmer Eye Institute, Department of Ophthalmology, University of Miami Miller School of Medicine, Miami, Florida, United States of America; 2 Department of Ophthalmology, College of Medicine, King Abdul Aziz University Hospital, King Saud University, Riyadh, Saudi Arabia; 3 Department of Microbiology and Immunology, University of Miami Miller School of Medicine, Miami, Florida, United States of America; National Institutes of Health, United States of America

## Abstract

Diabetic retinopathy is one of the main microvascular complications of diabetes and remains one of the leading causes of blindness worldwide. Recent studies have revealed an important role of inflammatory and proangiogenic high mobility group 1 (HMGB-1) cytokine in diabetic retinopathy. To elucidate cellular mechanisms of HMGB-1 activity in the retina, we performed this study. The histological features of diabetic retinopathy include loss of blood-vessel pericytes and endothelial cells, as well as abnormal new blood vessel growth. To establish the role of HMGB-1 in vulnerability of endothelial cells and pericytes, cultures of these cells, or co-cultures with glial cells, were treated with HMGB-1 and assessed for survival after 24 hours. The expression levels of the cytokines, chemokines, and cell adhesion molecules in glial and endothelial cells were tested by quantitative RT-PCR to evaluate changes in these cells after HMGB-1 treatment. Animal models of neovascularization were also used to study the role of HMGB-1 in the retina. We report that pericyte death is mediated by HMGB-1-induced cytotoxic activity of glial cells, while HMGB-1 can directly mediate death of endothelial cells. We also found that HMGB-1 affects endothelial cell activity. However, we did not observe a difference in the levels of neovascularization between HMGB-1-treated eyes compared to the control eyes, nor in the levels of proangiogenic cytokine VEGF-A expression between glial cells treated with HMGB-1 and control cells. Our data also indicate that HMGB-1 is not involved in retinal neovascularization in the oxygen-induced retinopathy model. Thus, our data suggest that retinal pericyte and endothelial injury and death in diabetic retinopathy may be due to HMGB-1-induced cytotoxic activity of glial cells as well as the direct effect of HMGB-1 on endothelial cells. At the same time, our findings indicate that HMGB-1 plays an insignificant role in retinal and choroidal neovascularization.

## Introduction

Diabetic retinopathy is the most common microvascular sequelae of diabetes and remains one of the leading causes of blindness worldwide among adults [1]–[3]. Diabetic retinopathy is characterized by gradually progressive alterations in the retinal microvasculature, leading to areas of retinal nonperfusion, increased vasopermeability, and pathologic intraocular proliferation of retinal vessels in response to retinal nonperfusion [1]–[3]. Due to progressive retinal capillary dropout, the ischemic retina mounts an angiogenic response leading to a more advanced form of the disease, proliferative diabetic retinopathy [1]–[3]. However, the exact molecular mechanism that mediates such a response remains largely unknown.

Ischemia is a common phenomenon in a retina suffering from diabetic retinopathy, which often leads to necrotic regions [2], [4]–[6]. High mobility group 1 (HMGB-1) protein is a nuclear DNA-binding protein released passively from necrotic cells as well as actively from monocytes/macrophages and endothelial cells [7]–[10]. Outside the cell, HMGB-1 can activate the pattern recognition receptors toll-like receptor 4 (TLR4) and receptor for advanced glycation end products (RAGE), triggering inflammation and damage as well as promoting angiogenesis in tissue [7]–[10]. The published literature demonstrated elevated levels of HMGB-1 in the vitreous fluid from patients suffering from PDR and increased HMGB-1 expression in the retinas of diabetic mice [11]–[15]. Therefore, HMGB-1 can rapidly leak out from the cells of ischemic regions of the diabetic retina, thus inducing angiogenesis. However, HMGB-1 pro-inflammatory toxicity can mediate damage of previously existing and new forming blood vessels as well as can promote infiltration of leukocytes, thereby increasing blood-retinal permeability in the diabetic retina. It was previously shown that in the diabetic retina, HMGB-1 initiates a pro-inflammatory response and disrupts the retinal vascular barrier [13], [15], [16]. To elucidate in which cell types HMGB-1 exerts its effects, we turned to the primary cell cultures. In this study we also tested the putative role of HMGB-1 in retinal and choroidal neovascularization.

## Materials and Methods

### Materials

All chemicals and reagents were purchased from Sigma-Aldrich (St. Louis, MO), Life Technologies (Grand Island, NY), and Thermo Scientific (Rockford, IL). Recombinant HMGB-1 was purchased from R&D Systems (Minneapolis, MN) and IBL International Corp (Toronto, ON).

### Animals

All experiments were performed in compliance with the NIH Guide for the Care and Use of Laboratory Animals and according to the University of Miami Institutional Animal Care and Use Committee approved protocols. C57BL/6J (stock number 000664) mice and Tlr4-deficient animals (stock number 007227) were obtained from the Jackson Laboratory (Bar Harbor, ME). Sprague-Dawley rats (2–3 months old) were purchased from Harlan Laboratories (Indianapolis, IN). Animals were housed under standard conditions of temperature and humidity, with a 12 hour light/dark cycle and free access to food and water.

### Mouse Model of Oxygen-induced Retinopathy

To induce retinopathy, postnatal day (p)7 mice, along with nursing females, were exposed to 75% oxygen for 5 days and then allowed to recover in room air on p12. Room air control litters were maintained under identical conditions as the hyperoxia-exposed mice. The hyperoxia-exposed and room air control pups were killed by CO_2_ euthanasia or cervical dislocation on p15 and p17.

### Primary Cell Cultures

Primary human vascular pericytes were obtained from ScienCell Research Laboratories (Carlsbad, CA). Primary human retinal microvascular endothelial cells were purchased from Cell Systems Corporation (Kirkland, WA). Pericytes and endothelial cells during the experiment were maintained in Dulbecco’s Modified Eagle’s Medium (DMEM) (Invitrogen, Carlsbad, CA) containing 0.1% heat-inactivated fetal bovine serum (FBS) (Invitrogen, Carlsbad, CA) and 1% antibiotic/antimycotic (Ab-Am) (Invitrogen, Carlsbad, CA). Astrocytes and microglial cells were prepared from the brains of neonatal (p3) mice as previously described [7]. To prepare pericytes/glia or endothelial cell/glia co-cultures, pericytes or endothelial cells were plated on cover slips in 24-well plates. Glia (50% astrocytes and 50% microglial cells) were plated in culture inserts and placed into the culture wells containing pericytes or endothelial cells 24 hours prior to treatment. Pericytes/glia and endothelial cell/glia co-cultures were maintained during experiment in DMEM containing 0.1% FBS (Invitrogen, Carlsbad, CA) and 1% Ab-Am (Invitrogen, Carlsbad, CA).

### Cell Death Assay

After treatment, necrotic and apoptotic cells were determined using a Vybrant Apoptosis Assay Kit (Invitrogen, Carlsbad, CA). Cells were imaged using a confocal microscope (Leica TSL AOBS SP5; Leica Microsystems). Individual glasses were sampled randomly to collect a total of 10 images using a 20× objective lens. The necrotic and apoptotic cells were counted using ImageJ software. The percentage of necrotic and apoptotic cells relative to the total number of cells was determined. The experiment was repeated at least five times.

### Quantitative RT-PCR Analysis

Quantitative RT-PCR analysis was performed using mouse and human gene-specific primers as previously described [17], [18] (Table 1). Specifically, RNA was extracted from glial cells or endothelial cells using RNA Nanoprep kit (Agilent Technologies, Santa Clara, CA), and reverse transcribed with the Reverse Transcription System (Promega, Madison, WI) to synthesize cDNA. Quantitative PCR was performed in the Rotor-Gene Q Cycler (Qiagen, Valencia, CA) using the SYBR GREEN PCR MasterMix (Qiagen, Valencia, CA). For each gene, relative expression was calculated by comparison with a standard curve, following normalization to the housekeeping gene β-actin (*ACTB*) expression chosen as a control.

**Table 1:** List of PCR primers.

Gene	*Mus musculus*	*Homo sapiens*
***TNF-α/***Forward	CAAAATTCGAGTGACAAGCCTG	GGCAGGTTCTCTTCCTCTCA
***TNF-α***/Reverse	GAGATCCATGCCGTTGGC	GCCAGAGGGCTGATTAGAGA
***IL-1β***/Forward	GACCTTCCAGGATGAGGACA	CAGCCAATCTTCATTGCTCA
***IL-1β/***Reverse	AGGCCACAGGTATTTTGTCG	GAACCAGCATCTTCCTCAGC
***IL-6/***Forward	ATGGATGCTACCAAACTGGAT	AGGAGACTTGCCTGGTGAAA
***IL-6/***Reverse	TGAAGGACTCTGGCTTTGTCT	AAAGCTGCGCAGAATGAGAT
***CCL2/***Forward	AGGTCCCTGTCATGCTTCTG	GCCTCCAGCATGAAAGTCTC
***CCL2/***Reverse	ATTTGGTTCCGATCCAGGTT	CATGGAATCCTGAACCCACT
***CCL5/***Forward	AGCAGCAAGTGCTCCAATCT	AACCCAGCAGTCGTCTTTGT
***CCL5/***Reverse	ATTTCTTGGGTTTGCTGTGC	TCCCAAAGTGCTGGGATTAC
***CXCL10/***Forward	GCTGCAACTGCATCCATATC	AGGAACCTCCAGTCTCAGCA
***CXCL10/***Reverse	CACTGGGTAAAGGGGAGTGA	TTCTTGATGGCCTTCGATTC
***NOX2/***Forward	GACTGCGGAGAGTTTGGAAG	
***NOX2/***Reverse	ACTGTCCCACCTCCATCTTG	
***NCF1/***Forward	CGAGAAGAGTTCGGGAACAG	
***NCF1/***Reverse	AGCCATCCAGGAGCTTATGA	
***NOS2/***Forward	CAGAGGACCCAGAGACAAGC	
***NOS2/***Reverse	TGCTGAAACATTTCCTGTGC	
***ICAM-1/***Forward		CCCTGTCAGTCCGGAAATAA
***ICAM-1/***Reverse		GATGACTTTTGAGGGGGACA
***TLR4/***Forward		TCAGAAACTGCTCGGTCAGA
***TLR4/***Reverse		GCCTCAGGGGATTAAAGCTC
***RAGE***/Forward		GATCCAGGATGAGGGGATTT
***RAGE***/Reverse		CAGGGTGTCTCCTGGTCTGT
***ACTB***/Forward	CACCCTGTGCTGCTCACC	AGCACAGAGCCTCGCCTTT
***ACTB***/Reverse	GCACGATTTCCCTCTCAG	GATGGGGTACTTCAGGGTGA

### Matrigel Induced Model of Neovascularization

Neovascularization was induced as previously described [19]. Briefly, HMGB-1 in phosphate buffered saline (PBS) was mixed with Matrigel (growth factor–reduced synthetic matrix; BD Biosciences, San Jose, CA) before injection. Matrigel, diluted with PBS only, was used as a control. Matrigel with HMGB-1 and PBS only was slowly injected to form a bleb (0.8 µl). The injecting needle was kept in place for 1–2 minutes for the gel to solidify before withdrawing. Blood vessels were visualized with a DiI solution 10 days after injection. At the end, animals were killed by CO_2_ overdose and perfused with PBS, followed by the DiI solution. Eyecups were obtained by removing the anterior segment of the eye and post-fixed in the same fixative overnight, rinsed with PBS, and embedded in 5% agarose. Serial sections (100 µm) covering the entire Matrigel area were cut on a vibratome (Leica Microsystems, Bannockburn, IL), mounted on glass slides with 80% glycerol, and examined by confocal microscopy.

### Western Blot Analysis

To collect vitreous humor (VH), the sclera was penetrated by using a 33G needle at a point temporal and posterior to the limbus, behind the lens. A small drop of VH that drained out was collected. This collected volume was equal to 2.5 µl, which was transferred to the tube containing 12.5 µl of PBS. The tube was centrifuged at 1100 RPM for 15 minutes at +4°C and the supernatant was collected. To evaluate the HMGB-1 level, 10 µl of the supernatant were loaded, and the proteins were size-separated in SDS PAGE gel. Proteins were blotted onto a PVDF membrane (Invitrogen, Carlsbad, CA) and incubated with HMGB-1 primary antibody (1∶1000, Abcam, Cambridge, MA). Proteins recognized by the antibody were revealed by an ultrasensitive chemiluminescent substrate system (SuperSignal West Femto, Thermo Scientific, Rockford, IL) according to instructions. Quantification of the protein bands was performed using image acquisition and analysis software (Quantity One; Bio-Rad Laboratories, Hercules, CA).

### Statistical Analysis

Statistical analysis was performed with one-way ANOVA followed by the Tukey test for multiple comparisons. In the case of single comparisons, the Student t-test was applied. P-values less than or equal to 0.05 were considered statistically significant.

## Results

### HMGB-1 can Affect Blood Retinal Permeability by Mediating Death of Blood-vessel Cells and Endothelial Cell Activity

**Figure 1:**
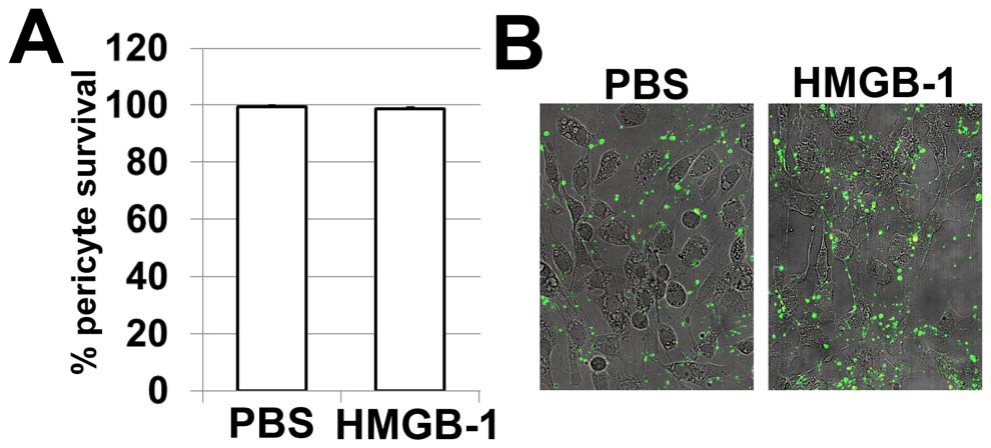
HMGB-1 does not directly mediate pericyte death. (**A**) Treatment with HMGB-1 (10 µg/ml) does not increase cell death in pure primary human pericyte cultures. All experiments were repeated at least three times. (**B**) Pericyte death was determined using annexin V as a marker of apoptotic cells and annexinV/propidium iodide (PI) to identify necrotic cells.

**Figure 2:**
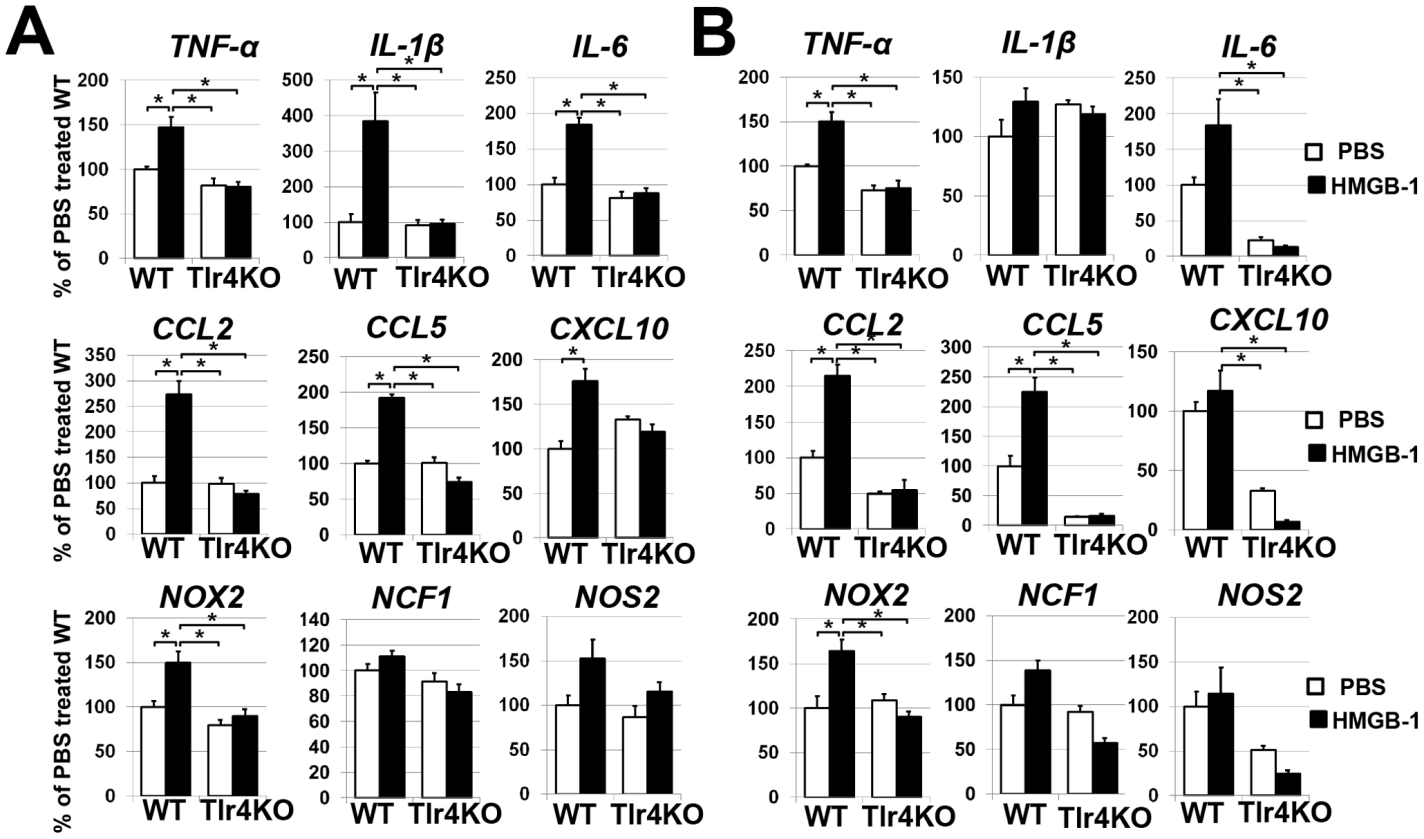
HMGB-1 induces expression of pro-inflammatory markers in astrocytes (A) and microglial cells (B) isolated from wild type (WT) animals. TLR4 deficiency (Tlr4KO) suppresses induction of pro-inflammatory markers after HMGB-1 treatment. Gene expression was assessed using quantitative RT-PCR in PBS treated controls and HMGB-1 treated cells. For each gene, results are expressed as a percentage of the corresponding value in the PBS treated primary glial cell cultures isolated from WT animals after normalization to β-actin (*P<0.05, n = 6).

**Figure 3:**
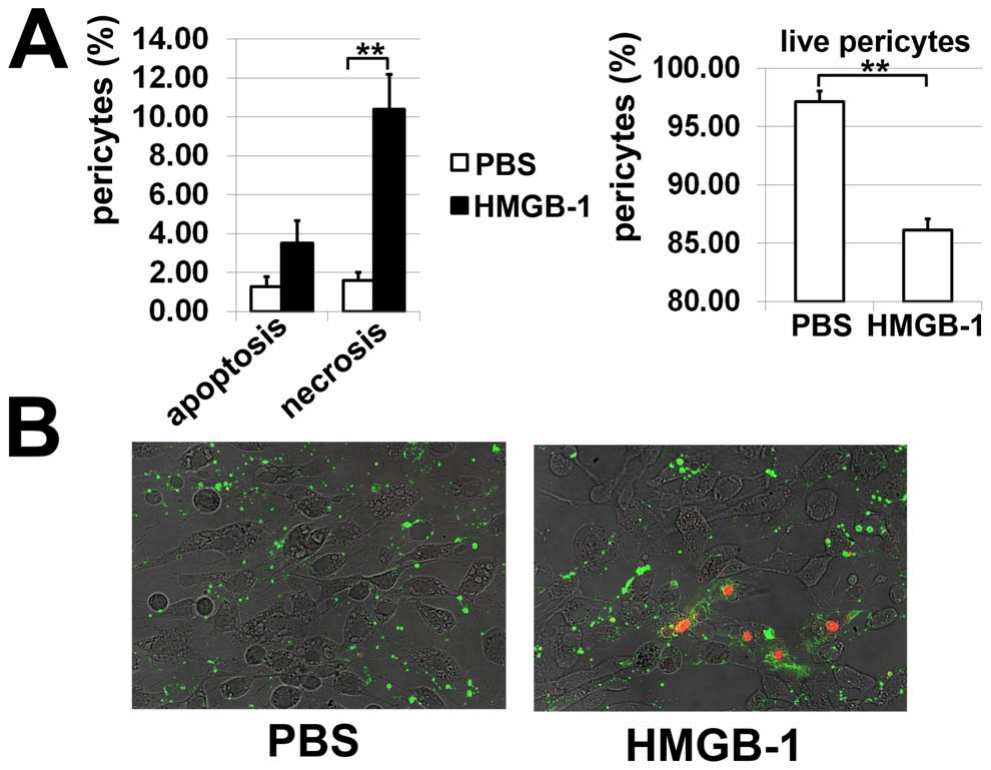
Glia mediates the effect of HMGB-1. (**A**) Primary pericytes co-cultured with glia were treated with HMGB-1 (5 µg/mL, 24 hours). Pericyte survival was assessed using an apoptosis assay kit. All experiments were repeated at least three times (**P<0.01). (**B**) Representative confocal images.

Blood-vessels become hemorrhagic and hyperdilated when they lose pericytes, the vessel support cells [20]. The loss of the pericytes is characteristic for the diabetic retina [1]–[3]. The published literature demonstrated elevated levels of HMGB-1 in diabetic retinas [11], [12], [15]. We demonstrated previously that HMGB-1 can mediate RGC death [7]. Since HMGB-1 receptors are present on pericytes [21], [22], we asked the question whether HMGB-1 promotes the death of these cells. To this end, we performed experiments with primary cultures of human pericytes to establish the direct role of HMGB-1 in the vulnerability of these cells. Pericytes were treated with HMGB-1 (10 µg/ml) or PBS (control) and then assessed for levels of necrotic, apoptotic, and live cells after 24 hours. Quantification of pericyte death did not show any difference between cultures treated with HMGB-1 versus PBS (Figure 1). It is known that pericytes interact with astrocytic foot processes to constitute the blood retinal barrier and microglial cells are present within all layers of the retina [20], [23]. Since glia were required for the harmful effect of HMGB-1 [7], we suggested that glial cells can mediate production of cytotoxic factors in the presence of HMGB-1 and thus promote death of pericytes. To test this hypothesis, we first evaluated whether glial cells that were exposed to HMGB-1 showed enhanced expression of cytotoxic markers. To this end, glial cells were treated with HMGB-1 (5 µg/ml) or PBS and the expression of the corresponding genes was evaluated after 24 hours by quantitative RT-PCR. We found increased expression of *TNF-α*, *IL-1β* and *IL-6* cytokines, and NOX2 NADPH oxidase subunits in HMGB-1 treated glial cells isolated from WT animals, while the levels of these genes were not significantly changed in TLR4-deficient treated and control glial cells (Figures 2A and 2B). Importantly, we found that glial cells treated with HMGB-1 significantly increased expression of *CCL2 (MCP-1)*, *CCL5 (RANTES)*, and *CXCL10* (*IP-10*) chemokines, and thus can promote leukocyte infiltration in the diabetic retina. Next, we evaluated pericyte survival in pericyte/glia co-cultures. Primary pericytes were plated on cover slips in 24-well plates. Glia were plated in culture inserts and placed into the culture wells containing pericytes 24 hours prior to treatment. These co-cultures were challenged with HMGB-1 (5 µg/ml) or PBS and the levels of living pericytes were detected after 24 hours. We found that treatment with HMGB-1 decreased the number of living pericytes in the presence of glial cells (Figure 3). Thus, these data suggest that pericyte death was indirectly mediated by HMGB-1-induced cytotoxic activity of glial cells. This activity can be mediated, at the very least, due to the glial TLR4 signaling cascade.

**Figure 4:**
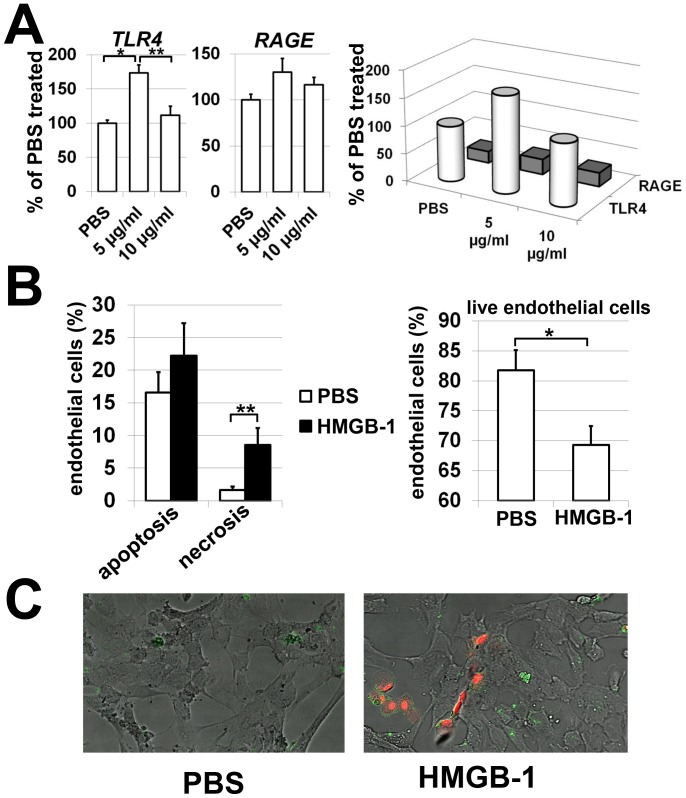
HMGB-1 directly mediates retinal endothelial cell death. (**A**) Expression of HMGB-1 receptors (TLR4 and RAGE) in HMGB-1-treated (5 and 10 µg/ml) and control (PBS-treated) retinal endothelial cells. (**B**) Treatment with HMGB-1 (10 µg/ml) increases cell death in primary human retinal endothelial cell cultures. All experiments were repeated at least three times (*P<0.05, **P<0.01). (**C**) Endothelial cell death was determined using annexin V and annexinV/PI to identify apoptotic and necrotic cells, respectively.

**Figure 5:**
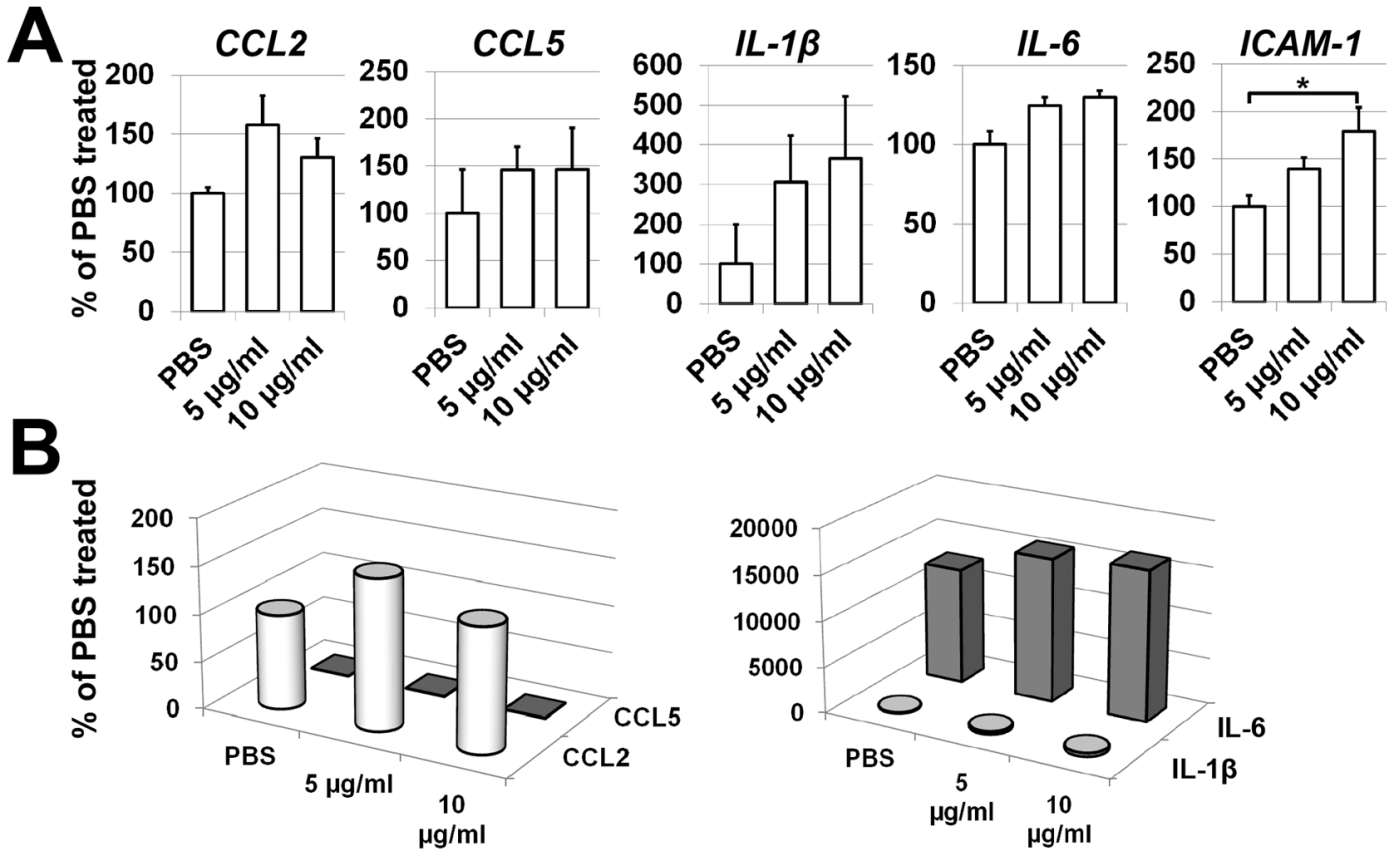
HMGB-1 can affect retinal endothelial cell activity. (**A**) Expression of *CCL2* and *CCL5* chemokines, *IL-1β* and *IL-6* cytokines, as well as cell adhesion molecule *ICAM-1* in HMGB-1-treated (5 and 10 µg/ml) and control (PBS-treated) retinal endothelial cells. Gene expression was assessed using quantitative RT-PCR in cells exposed to HMGB-1 or PBS after 24 hours (*P<0.05, **P<0.01, n = 6). (**B**) Comparative analysis of chemokine and cytokine expression in HMGB-1 treated retinal endothelial cells.

Death of retinal endothelial cells in the diabetic retina has been demonstrated *in vivo* and ultimately results in the formation of acellular capillaries [24]. Since HMGB-1 could also affect endothelial cell survival in the diabetic retina, we tested primary retinal endothelial cells treated with HMGB-1. First, we evaluated expression of HMGB-1 receptors (TLR4 and RAGE) in HMGB-1 and PBS (control) -treated cells. The changes in expression of TLR4 and RAGE were assessed 24 hours after treatment by quantitative RT-PCR. We found increased levels of TLR4 expression in HMGB-1 treated cells, but did not observe statistically significant changes in RAGE expression (Figure 4A). Importantly, the level of TLR4 in treated and control retinal endothelial cells was significantly higher compared to RAGE (Figure 4A). Since HMGB-1 receptors were present on retinal endothelial cells, we next assayed survival of these cells after HMGB-1 treatment. Primary retinal endothelial cells were treated with HMGB-1 (10 µg/ml) or PBS and then assessed for levels of necrotic and apoptotic cells and survival after 24 hours. We observed higher levels of retinal endothelial cell death in cultures treated with HMGB-1 than with PBS (Figure 4B, 4C). Thus, our data indicate that HMGB-1 can directly mediate death of retinal endothelial cells. Next, we asked the question whether HMGB-1 affects endothelial cell activity, since it is changed significantly in the diabetic retina and associated with increased blood retinal permeability [1], [2], [25]. To this end, primary retinal endothelial cells were treated with HMGB-1 (5 and 10 µg/ml) or PBS (control) and the expression of the chemokines, cytokines, and cell adhesion molecules was evaluated after 24 hours by quantitative RT-PCR. We detected high levels of *CCL2* chemokine in PBS treated cultures (Figure 5). The levels of *CCL2* and *CCL5* expression were slightly increased in HMGB-1 treated cells (Figure 5). At the same time, we did not detect *CXCL10* expression in treated or control cells. We also found high *IL-6* expression in control cells, which was slightly increased in HMGB-1-treated cultures (Figure 5). We did not observe *TNF-α* expression in treated or control cells. We also noted that the larger value of *IL-1β* expression was one hundred times less than the smaller value of *IL-6* expression (Figure 5). We found slightly increased expression of *ICAM-1* in HMGB-1-treated cells (Figure 5). Thus, since HMGB-1 is present in the diabetic retina, it can also affect retinal endothelial cell activity.

### HMGB-1 Plays Insignificant Role in Retinal and Choroidal Neovascularization

**Figure 6:**
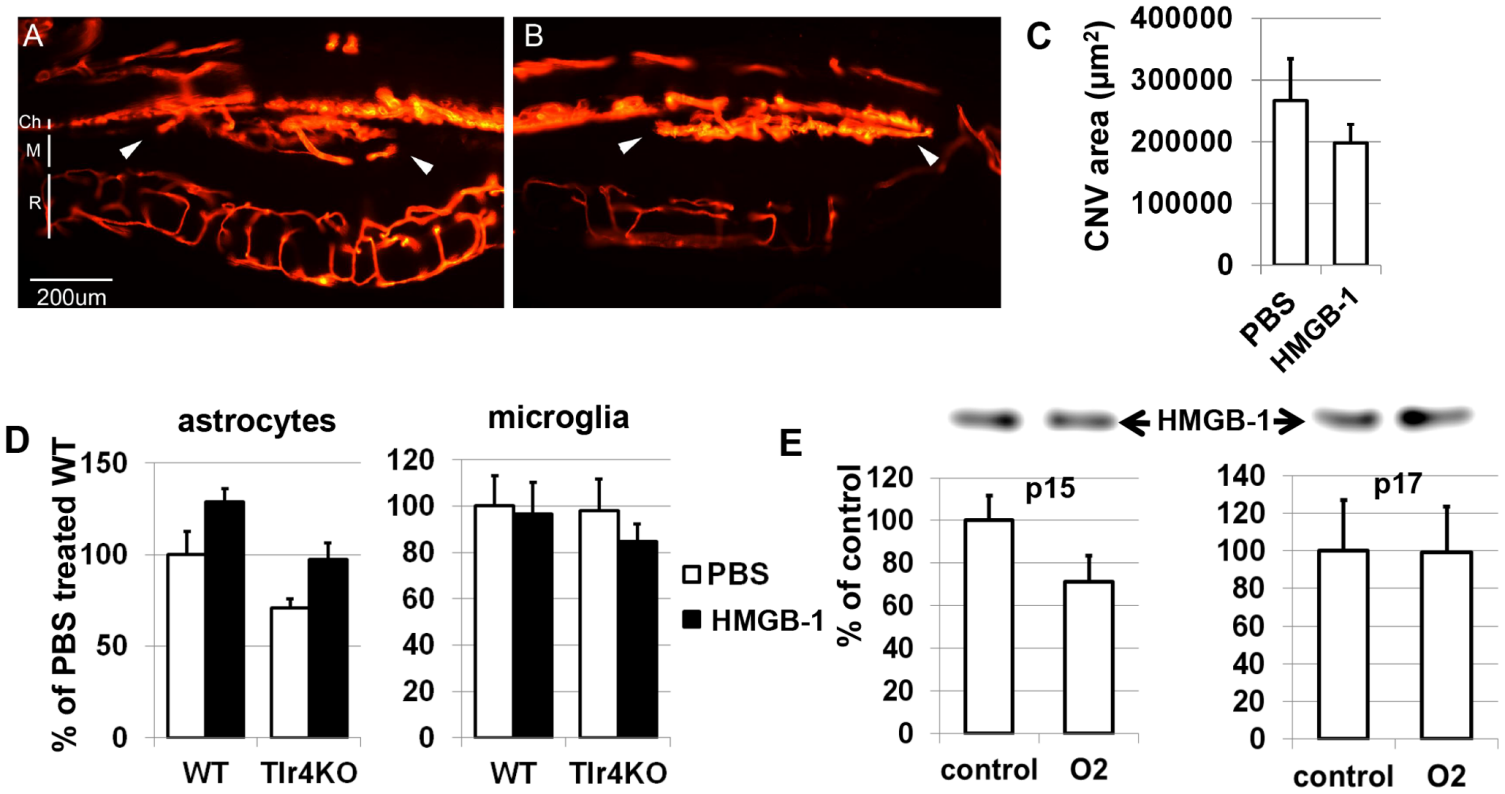
There was no association found between the level of HMGB-1 and neovascularization in the retina. Neovascularization was induced by subretinally injecting Matrigel either with PBS (**A**) or mixed with HMGB-1 (**B**). Neovascularization was allowed to develop for 10 days. Blood vessels were labeled with DiI and visualized by fluorescence microscopy (white arrows indicate neovascularization in the Matrigel injected area. Ch, choroids; M, Matrigel, R retina. Scale bar: 200 µm). (**C**) Quantitative analysis shows no difference in the levels of neovascularization between HMGB1-treated eyes compared to control eyes. (**D**) *VEGF-A* expression was not changed in glial cells (astrocytea and microglia) treated with HMGB-1 and PBS (control). (**E**) Examination by western blot of vitreous humor from both hyperoxia-injured and room air control animals (obtained from postnatal days 15 and 17 pups) did not show a statistically significant difference between them. Results of analysis of western blots (top) are expressed as a percentage of corresponding value in the control eyes ± SEM (n = 6).

It was previously shown that extracellular HMGB-1 can mediate angiogenesis in tissues [9]. To test whether HMGB-1 regulates choroidal angiogenesis, subretinal injections of Matrigel mixed with HMGB-1 (1.25 µg in 0.8 µl) were performed. Matrigel mixed with PBS was injected to the contralateral eye and was used as a control. Blood vessels were visualized with a DiI solution on day 10 after injection [26]. Extensive neovascular networks from the choriocapillaries had developed in the Matrigel area by 10 days in all eyes (Figures 6A and 6B). However, we did not find a difference in the levels of neovascularization between HMGB-1 treated eyes compared to control eyes on day 10 (Figure 6C). Since HMGB-1 can induce angiogenesis by stimulating the secretion of proangiogenic cytokine VEGF-A, we decided to test *VEGF-A* expression by glial cells treated with HMGB-1. To this end, glial cells were treated with HMGB-1 (5 µg/ml) or PBS and the expression of the *VEGF-A* was evaluated after 24 hours by quantitative RT-PCR. We did not detect any difference in *VEGF-A* expression between glial cells treated with HMGB-1 versus those treated with PBS (Figure 6D). Finally, to evaluate the role of HMGB-1 in retinal angiogenesis, we turned to a mouse model of oxygen-induced retinopathy [27]. In this model, 7-day-old pups and their nursing mothers are subjected to hyperoxia (75% oxygen) for 5 days, a condition that inhibits retinal vessel growth and causes significant vessel loss. On postnatal day 12, the mice are returned to room air, and the hypoxic avascular retina triggers both normal vessel re-growth and retinal neovascularization, which is maximal at postnatal day 17. We postulated that if HMGB-1 is involved in retinal angiogenesis, we should observe increased levels of HMGB-1 in the vitreous humor of treated animals compared to control animals. To this end, experimental mice were exposed to oxygen concentrations of 75 ± 2% from postnatal days 7 to 12 and then allowed to recover in room air. Control animals were raised in room air. Eyes obtained from postnatal days 15 and 17, from both hyperoxia-injured and room air control animals were processed for vitreous humor examination by using western blot analysis. The western blot analysis of the vitreous humor obtained from p15 and p17 mice did not show a difference between treated and control animals (Figure 6E). These data suggest that HMGB-1 plays insignificant, if any, roles in mediating retinal neovascularization in the oxygen-induced retinopathy model. Taken together, these data suggests that HMGB-1 plays an insignificant role in retinal and choroidal neovascularization.

## Discussion

Diabetic retinopathy is one of the main microvascular complications of diabetes and one of the most common causes of blindness in people over the age of 50 [1]–[3]. The histological features of diabetic retinopathy include loss of blood-vessel pericytes and endothelial cells, and abnormal new blood vessel growth on the surface of the retina in the more advanced stage of the disease, proliferative diabetic retinopathy [1]–[3]. Recent studies have elucidated that HMGB-1 is involved in the pathogenesis of diabetic microvascular complications, including diabetic retinopathy [9], [11]–[16]. However, cellular mechanisms of HMGB-1 action in the diabetic retina remain incompletely understood. HMGB-1, a DNA-binding nuclear protein, can be released actively after cytokine stimulation and passively during necrotic cell death [7]–[10]. HMGB-1 can promote inflammatory responses and cause damage by numerous mechanisms [7]–[10], [28]. It was also shown that HMGB-1 can promote angiogenesis in some tissues [9], [29], [30]. In this study, we demonstrated that HMGB-1 can mediate endothelial cell death directly, while pericyte death was indirectly mediated by HMGB-1-induced cytotoxic activity of glial cells. HMGB-1 can also affect endothelial cell activity. However, our findings suggest that HMGB-1 plays an insignificant role in retinal and choroidal neovascularization.

Loss of pericytes, which provide vascular stability and control endothelial proliferation, is characteristic for the diabetic retina [31]. Receptors of HMGB-1 (RAGE and TLR4) are expressed in microvascular pericytes [21], [22] and thus, can promote HMGB-1 toxicity in the diabetic retina. Surprisingly, we did not detect any difference in cell survival between PBS treated (control) primary pericytes and pericytes treated with high levels of HMGB-1 (10 µg/ml). Since pericytes are in close contact with glial cells and glia were required for the harmful effect of HMGB-1 [7], we tested the role of glial cells (astrocytes and microglia) in pericyte survival. Our findings suggest that HMGB-1-induced cytotoxic activity of glial cells can contribute to pericyte death due to cytokine activity and reactive oxygen species production in a TLR4-dependent manner. Importantly, we found an increased expression of chemokines that can facilitate leukocyte infiltration in the diabetic retina. Correlations between elevated numbers of accumulated leukocytes and capillary damage have been shown previously in the diabetic retina [25]. Thus, HMGB-1-dependent production of cytotoxic factors by glial cells can mediate pericyte death in the diabetic retina. HMGB-1-mediated production of chemokines by glial cells can make the environment even worse in the diabetic retina due to increased leukocyte infiltration. In addition to loss of pericytes, endothelial cell death is a hallmark of diabetic retinopathy [24], [31]. However, the mechanisms underlying diabetic retinal endothelial cell death remain largely unknown. Our study suggest that HMGB-1 can directly mediate retinal endothelial cell death and thus can promote the formation of acellular capillaries, lesions that are characteristic for the diabetic retina [24], [31]. The study of gene expression has also revealed that HMGB-1 affects endothelial cell activity. We found slightly increased expression of *CCL2* (MCP-1) chemokine and the *ICAM-1* cell adhesion molecule in HMGB-1 treated endothelial cells. HMGB-1-dependent increased expression of chemokines by glial cells and endothelial cells can attract leukocytes to the diabetic retina [25]. Increased expression of ICAM-1 can facilitate increased adhesion of leukocytes to endothelia [25]. It was shown that adherent leukocytes mediate endothelial cell injury and death in the diabetic retina [25], [32]. These data suggest that HMGB-1 can also indirectly mediate endothelial cell death in patients suffering from diabetic retinopathy.

Most of the diabetes-related vision loss is caused by complications from abnormal retinal blood vessel growth [33]. Since HMGB-1 can promote neovascularization in many tissues [9], [29], [30], we tested whether HMGB-1 mediates neovascularization in the retina. We evaluated this possibility in various ways. First, we subretinally injected high levels of HMGB-1, but did not detect any difference in the levels of neovascularization between HMGB-1 treated eyes compared to control eyes. We also tested HMGB-1 levels in the vitreous humor of control animals and animals with oxygen-induced retinal neovascularization, model of oxygen-induced retinopathy [27]. We hypothesized that if HMGB-1 is involved in retinal neovascularization in this model, we should observe an increased level of HMGB-1 in the vitreous humor of treated animals as compared to the control animals. Since we did not observe any difference in the levels of HMGB-1 between the control and experimental groups of animals, we suggested that retinal neovascularization in this model is HMGB-1 independent or that the role of HMGB-1 in retinal neovascularization is insignificant. Since vascular endothelial growth factor (VEGF-A) induces neovascularization in the diabetic retina [34], we decided to evaluate *VEGF-A* expression in HMGB-1 treated cells. Most types of cells, excluding endothelial cells themselves, produce VEGF-A. In this study, we used glial cells to test the ability of HMGB-1 to affect *VEGF-A* expression. Unfortunately, we did not observe statistically significant changes in *VEGF-A* expression. However, the results of the Biscetti and colleagues study showed that HMGB-1 mediates ischemia-induced angiogenesis in diabetic mice in a VEGF-A-dependent manner [29]. Considering these data together, we suggested that HMGB-1 cannot mediate angiogenesis in the retina by itself. Additional factors may be required to initiate HMGB-1-dependent neovascularization in the retina.

Finally, in this study, we investigated cellular mechanisms of HMGB-1 action in the diabetic retina. Our findings indicate that retinal pericyte and endothelial injury and death in diabetic retinopathy may be due to HMGB-1-induced cytotoxic activity of glial cells as well as the direct effect of HMGB-1 on endothelial cells. At the same time, our data suggest that HMGB-1 plays an insignificant role in retinal and choroidal neovascularization. Thus, this study provides further insight into the role of HMGB-1 in diabetic retina and may provide therapeutic opportunities for patients suffering from diabetic retinopathy.
